# The microbiome of the Black Sea water column analyzed by shotgun and genome centric metagenomics

**DOI:** 10.1186/s40793-021-00374-1

**Published:** 2021-03-16

**Authors:** Pedro J. Cabello-Yeves, Cristiana Callieri, Antonio Picazo, Maliheh Mehrshad, Jose M. Haro-Moreno, Juan J. Roda-Garcia, Nina Dzhembekova, Violeta Slabakova, Nataliya Slabakova, Snejana Moncheva, Francisco Rodriguez-Valera

**Affiliations:** 1 Evolutionary Genomics Group, Departamento de Producción Vegetal y Microbiología, Universidad Miguel, Hernández, San Juan de Alicante, Alicante, Spain; 2 National Research Council (CNR), Institute of Water Research (IRSA), Verbania, Italy; 3 Cavanilles Institute of Biodiversity and Evolutionary Biology, University of Valencia, E-46980 Paterna, Valencia Spain; 4 Department of Aquatic Sciences and Assessment, Swedish University of Agricultural Sciences, Lennart Hjelms väg 9, 75651 Uppsala, Sweden; 5 Institute of Oceanology “Fridtjof Nansen” – Bulgarian Academy of Sciences, Varna, Bulgaria; 6 Moscow Institute of Physics and Technology, Dolgoprudny, 141701 Russia

**Keywords:** Black Sea microbiota, Genome-resolved metagenomics, Redoxcline, Euxinic waters

## Abstract

**Background:**

The Black Sea is the largest brackish water body in the world, although it is connected to the Mediterranean Sea and presents an upper water layer similar to some regions of the former, albeit with lower salinity and temperature. Despite its well-known hydrology and physicochemical features, this enormous water mass remains poorly studied at the microbial genomics level.

**Results:**

We have sampled its different water masses and analyzed the microbiome by shotgun and genome-resolved metagenomics, generating a large number of metagenome-assembled genomes (MAGs) from them. We found various similarities with previously described Black Sea metagenomic datasets, that show remarkable stability in its microbiome. Our datasets are also comparable to other marine anoxic water columns like the Cariaco Basin. The oxic zone resembles to standard marine (e.g. Mediterranean) photic zones, with Cyanobacteria (*Synechococcus* but a conspicuously absent *Prochlorococcus*), and photoheterotrophs domination (largely again with marine relatives). The chemocline presents very different characteristics from the oxic surface with many examples of chemolithotrophic metabolism (*Thioglobus*) and facultatively anaerobic microbes. The euxinic anaerobic zone presents, as expected, features in common with the bottom of meromictic lakes with a massive dominance of sulfate reduction as energy-generating metabolism, a few (but detectable) methanogenesis marker genes, and a large number of “dark matter” streamlined genomes of largely unpredictable ecology.

**Conclusions:**

The Black Sea oxic zone presents many similarities to the global ocean while the redoxcline and euxinic water masses have similarities to other similar aquatic environments of marine (Cariaco Basin or other Black Sea regions) or freshwater (meromictic monimolimnion strata) origin. The MAG collection represents very well the different types of metabolisms expected in this kind of environment. We are adding critical information about this unique and important ecosystem and its microbiome.

**Supplementary Information:**

The online version contains supplementary material available at 10.1186/s40793-021-00374-1.

## Background

The Black Sea is the innermost arm of the Mediterranean basin. Nearly severed from the rest by the tectonic movement of the African plate, it is only connected to the Mediterranean Sea by the narrow but deep strait of the Bosporus. The Black Sea has a positive hydric balance i.e. receives more freshwater than lost by evaporation and hence contains less salt (from 0.73% in epipelagic to 2.2% in meso-bathypelagic waters) than the Mediterranean proper (3.8%). In addition, the large watershed and riverine inputs lead to a richer nutrient status (meso-eutrophic) and permanent stratification with a colder, more saline deepwater mass that remains anaerobic and largely euxinic below 150–200 m [1–3]. All these properties make the Black Sea a unique brackish-marine environment. Its depth (average 1253 m with a maximum of 2212 m) makes this system much more stable than other brackish inland water bodies like the Baltic Sea in which the anaerobic compartment is only a recent development due to anthropogenic impacts [4].

Although a few studies have been carried out by metagenomics and metagenome-assembled genomes (MAGs) reconstruction, the information available in databases about this unique environment is scarce. A recent study showed for the first time the microbial structure of the sulfidic waters of 1000 m depth [5], mainly dominated by sulfate reducers (*Desulfobacterota* and *Chloroflexi* classes such as *Dehalococcoidia* or *Anaerolinea*), associated dissolved organic matter (DOM) degraders (*Marinimicrobia*, *Cloacimonadota*) and streamlined uncultured taxa such as *Omnitrophota*, *Parcubacteriota* or *Woesearchaeota* [6]. At the genome level, Black Sea microbes remain largely unknown. Only 10 MAGs were studied in the abovementioned work [5]. However, 179 MAGs from 50 to 2000 m have been recently deposited into Genbank (PRJNA649215) alongside two recent works that have described various *Synechococcus* phylotypes [7, 8]. Most recently, members of widespread clades such as SUP05 (Ca. Thioglobus spp), *Sulfurimonas* bacteria, and uncultivated SAR324 and Marinimicrobia have been studied from this and other dysoxic environments [9]. Another habitat that could be comparable to the Black Sea in terms of water column stratification is the Cariaco Basin (Venezuela). Several studies on the redoxcline and euxinic strata on this system provided some of the first insights into microbial structuring in anoxic marine waters and Oxygen Minimum Zones (OMZs) [10, 11]. Here, we present a genome resolved metagenomic study of different depths in the Black Sea adding a total of 359 high-quality MAGs. The epipelagic and DCM strata show an overall marine-brackish community composition with a predominance of microbes similar to the Mediterranean and the Caspian Seas [12, 13]. The anaerobic compartment, which accounts for up to 80% of the total Black Sea volume, had a much more poorly understood microbiota including various members of the microbial “dark matter”.

## Results

### Black Sea shotgun metagenome analysis

**Fig. 1:**
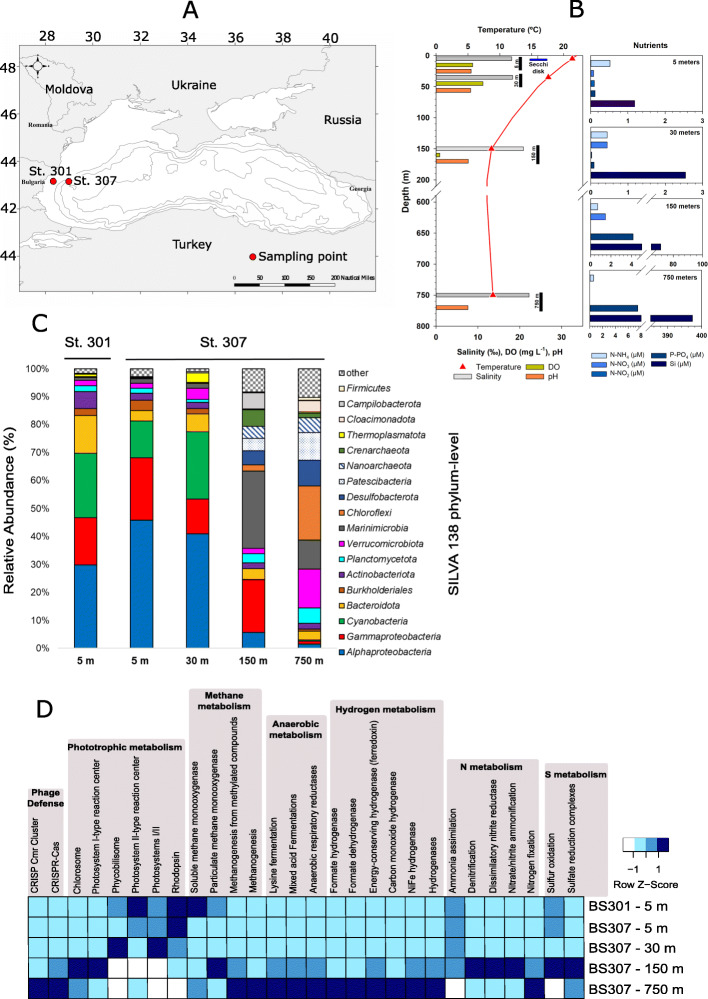
Black Sea sampling, physicochemical parameters and raw reads analysis. **a** A map of the Black Sea basin showing the red circle-coded sampling points of this study. **b** Bar plot showing the different environmental parameters and physicochemical profiles measured. Each environmental measurement is color-coded. **c** Black Sea phylum level 16S rRNA gene taxonomic classification assessed with unassembled reads individually mapped into SILVA138 database. **d** Metabolic profiles assessed with abundance values obtained from reads and SEED subsystems and pathways, pecific proteins/genes or microbial systems. A blue row Z-score scale was applied to statistically assess abundance values differing between samples

Samples were collected along the Bulgarian coast at two stations (Fig. 1a). Sampling depths were chosen on the grounds of the physicochemical measurements (Fig. 1b) to cover representative temperature, oxygen, and chlorophyll-a values (Additional File 1). Thus, for St. 307, with a maximum depth of 1100 m, samples were from the near-surface at 5 m depth, the deep chlorophyll maximum (DCM) at 30 m, a sample from the redoxcline/pycnocline at 150 m, and finally, a sample at 750 m, corresponding to the euxinic water layer. Additionally, we collected a single near-surface sample (5 m) closer to the shore at station 301 with a maximum depth of 22.5 m to examine the coastal influence, but no significant difference was found with the surface offshore sample, and hence this sample will not be described any further although it was used to generate 87 MAGs.

**Fig. 2:**
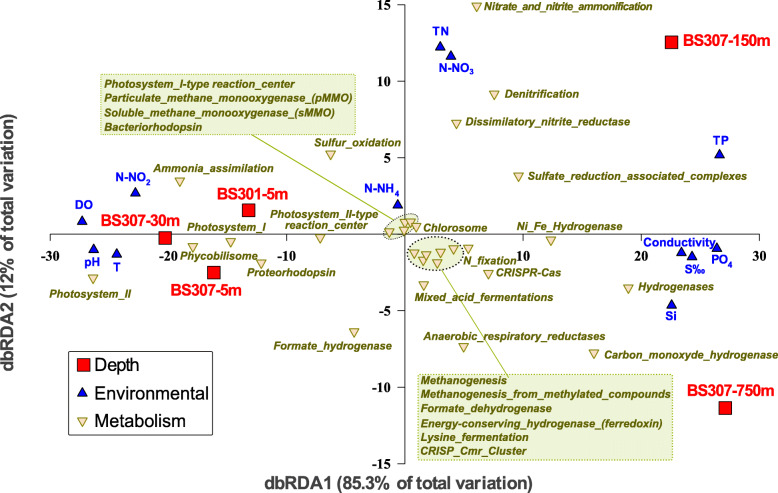
Black Sea distance-based redundancy analysis (dbRDA) between different samples (depths), environmental parameters and the metabolic pathways assessed by read abundance from SEED subsystems. Each Black Sea sample is red square-coded. Environmental parameters are blue triangle-coded an SEED pathways are cream triangle-coded

We first performed an unassembled read analysis for each depth to obtain an overview of the taxonomic profile based on 16S rRNA gene fragments retrieved from the metagenomes that were compared to the SILVA database [14] (Fig. 1c) and the main predicted metabolic functions assessed by the SEED subsystems [15] (Fig. 1d). We have also made a distance-based redundancy analysis (dbRDA) to statistically assess the correlation among samples, environmental parameters, and the abundance of metabolic pathways (Fig. 2).

The oxic strata (surface and DCM) presented, at this large-grained level, a very similar taxonomic composition. We have used throughout the taxonomic nomenclature derived from the Genome Taxonomy Database (GTDB) [16]. In the photic zone (5 m and DCM), *Alphaproteobacteria* (orders SAR11, SAR116, *Rhodobacterales,* and *Rhodospirillales*), *Gammaproteobacteria* (mostly orders SAR86 and *Pseudomonadales*), and picocyanobacteria (order *Synechococcales*) were the most abundant groups, representing > 70% of the microbial community (assessed by 16S rRNA gene fragment abundance). It must be highlighted the complete absence of the genus *Prochlorococcus* that contrastingly predominates in the Eastern Mediterranean Sea [12]. This absence has been assessed through genome recruitment of all *Prochlorococcus* genomes available in the NCBI thus far, having obtained no significant mapped reads at > 70% identity and > 50 bp of alignment lengths. The predominant metabolic subsystems of the oxic layer (Fig. 1d) were, as expected, associated with phototrophic lifestyles such as those from *Synechococcales* (photosystems/phycobilisomes) or photoheterotrophy with type-1 rhodopsin pumps (generally present in SAR11, SAR86, or *Flavobacteriales*). Besides, ammonia and nitrite were the preferred N source, whilst DO, T, and pH were the main environmental variables correlating to oxic samples (Fig. 2).

The taxonomic composition derived from 16S rRNA gene fragments changed dramatically as we reached oxygen extinction in the redoxcline (150 m), where various taxa and microbial lifestyles coexisted, with a prevalence of anaerobic N and S related subsystems (Fig. 1d and Fig. 2). *Marinimicrobia* (ca. 30% of 16S rRNA gene assigned reads) and *Gammaproteobacteria* (ca. 20%) were the dominant taxa (Fig. 1c). Also, significant percentages of various associated streamlined microbes such as *Patescibacteria* and *Nanoarchaeota* (ca. 4% each) were found in this layer. The redoxcline of the Black Sea presented the most metabolically diverse set of pathways among all analyzed samples (Fig. 1d and Fig. 2). The main environmental variables that were statistically grouped with the redoxcline were total nitrogen (TN) and nitrate, which were associated with the different N biogeochemical cycle pathways. The highest abundance pathways were associated with denitrification (nitrogen gas as the final product), nitrate/nitrite ammonification, and dissimilatory nitrate reduction (with ammonium as the final product), but the N cycle was also completed with ammonia oxidation and N fixation pathways detected both in total reads (Fig. 2) and MAGs (see below). Nonetheless, various other metabolisms coexisted in this thin layer where oxygen disappears. Such pathways, assigned from global read abundance included fermentation and hydrogen metabolism, chemolithotrophic, anoxygenic photosynthesis, or sulfate reduction.

The euxinic waters at 750 m had an expected increase in sulfate reducers (*Desulfobacterota*), *Dehalococcoidia/Anaerolineae Chloroflexota.* There were representatives from *Omnitrophota* and *Kiritimatiellae* (both classified inside *Verrucomicrobiota* according to SILVA standards [14], although *Omnitrophota* is classified as a single phylum according to GTDB [16]), *Phycisphaerae Planctomycetota, Marinimicrobia, Nanoarchaeota, Patescibacteria, and Cloacimonadota*. Finally, *Halobacterota* (*Syntrophoarchaeia*) and *Crenarchaeota* (*Bathyarchaeia*) minor representation (< 2% of total microbial biomass assessed by 16S rRNA gene) showed that methanogenesis coexisted with sulfate reduction in these euxinic waters, if in a much more reduced fraction. As for the SEED subsystems, the euxinic waters (750 m) showed an increase in fermentation, hydrogen metabolism, anaerobic respiratory reductases, or methanogenesis pathways (Fig. 1d and Fig. 2). Overall, we observed a huge diversity of microbiota providing hydrogen and fermentation by-products that conformed a syntrophic network fueling the sulfate reducers at the redox end.

### Comparison of the Black Sea and Cariaco Basin metagenomic datasets

To assess the representativity of our samples we compared the community structure by 16S rRNA gene (following the same abovementioned methodology) and the read similarity (clustering) among various similar datasets, including other Black Sea metagenomes available (PRJNA649215) [9] (see Fig. S1) and with a former sampling campaign from another marine euxinic system, the Cariaco Basin [10, 11] (see Fig. S2).

As depicted from Fig. S1, we have observed both a clear 16S rRNA gene taxonomic profile (Fig. S1A) and read clusterization (Fig. S1B) between Black Sea samples obtained from different sampling campaigns. It is remarkable the stability observed in this system, even though samplings were taken 6 years apart, and in different locations. All the abovementioned taxa showed up in the compared samples as well, observing a clear sample clusterization between our DCM sample at 30 m and a 50 m former sample (PRJNA649215). Our redoxcline (150 m) sample appears in between 130 and 250 m samples from PRJNA649215, whilst the mesopelagic euxinic 750 m sample that we took clusters with the group of former meso/bathypelagic strata (500, 1000, and 2000 m).

Regarding the Cariaco Basin (Venezuela), we compared exclusively redoxcline metagenomes from this marine environment with our two redoxcline and anoxic datasets, observing a markedly different taxonomic profile (Fig. S2A) and read clusterization (Fig. S2B). As a general trend, *Gammaproteobacteria*, SAR324, *Actinobacteriota,* and *Thermoplasmatota* showed a higher % in the Cariaco Basin. The most similar samples to our (150 m) in terms of taxonomy were the 247 and 314 m.

### Recovery of MAGs

**Fig. 3:**
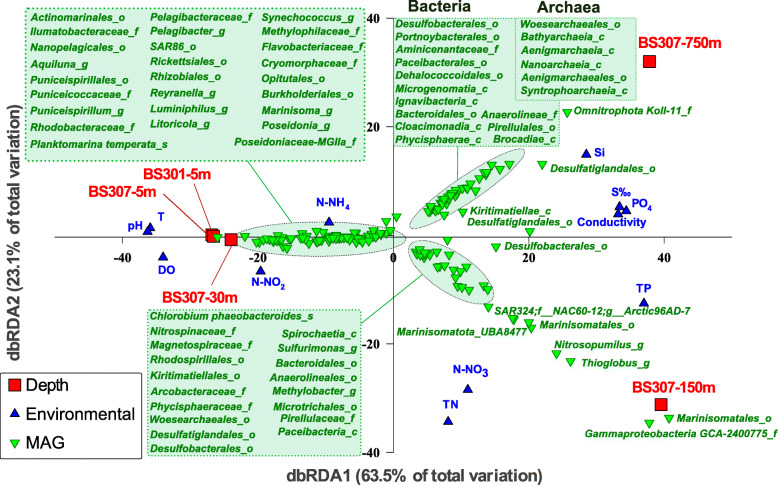
Black Sea Distance-based redundancy analysis (dbRDA) between different samples (depths), environmental parameters and MAGs abundance values obtained from the RPKGs.. We provided MAG names for class (_c), order (_o), family (_f), genus (_g) or species (_s) according to the last GTDB classification scheme followed in Table 1 and Table 2. Each Black Sea sample is red square-coded. Environmental parameters are blue triangle-coded and MAGs are green triangle-coded

Next, we have reconstructed metagenome-assembled genomes (MAGs) from each dataset separately. Automated binning followed by manual curation of the bins (see methods) generated 359 MAGs with > 50% completeness and < 5% contamination. Statistics of these MAGs are described in Table 1 (MAGs from oxic samples) and Table 2 (redoxcline/anoxic MAGs) and in individual detail in Additional File 2. We also plotted the estimated genome size versus GC content of all our MAGs in Fig. S3. To specifically determine which species dominated the different strata, as assessed above from the phylum level taxonomic 16S rRNA gene profile, we mapped the metagenomic reads into our MAGs. This relative abundance was assessed by read recruitment (> 95% of identity and > 50 bp of alignment lengths) and expressed as reads per Kb of Genome per Gb of metagenome (RPKGs) as shown in Additional File 3. Based on MAG abundance, we conducted another dbRDA analysis to assess sample and environmental parameter correlations (Fig. 3), which pictured 3 clusters of MAGs grouping with oxic samples (5 and 30 m), redoxcline (150 m), and euxinic (750 m) strata. To estimate the binning efficiency, we mapped the reads of each metagenome (same thresholds as above) against the MAGs obtained for each sample. The percentages of reads mapped to the MAGs varied among samples, being maximum in the redoxcline (50%), and minimum in the euxinic sample (33%). Concerning the oxic samples, the MAG recovery efficiency was ca. 50% of the total reads mapped with the MAGs from the coastal epipelagic sample (BS301–5 m), 42% for the off-shore epipelagic sample (BS307–5 m), and 34% for the DCM sample (BS307–30 m). These recovery values are in the range of what was previously obtained for other aquatic environments such as Lake Baikal [17].

**Table 1:** Summary statistics and features of Black Sea MAGs retrieved from 5 and 30 m samples

Phylum/Division	Taxonomic affiliation of MAGs (GTDB, Referenced groups)	n° of MAGs	Range Estimated Genome size (Mp)	Range GC (%)	Range median intergenic spacer (bp)	Av. Compl. (%)	Av. Cont. (%)
*α-Proteobacteria*	*g_Planktomarina (****3****), g_Puniceispirillum (****6****), g_Reyranella (****1****), f_Rhodobacteraceae (****7****), o_Pelagibacterales (****8****), o__Parvibaculales (****5****), f_Puniceispirillaceae (****6****), f_Nisaeaceae (****1****), o_Rickettsiales (****3****), o_Rhizobiales (****2****), o_Rhodospirillales_A (****1****), c_Alphaproteobacteria (****3****)*	52	1–7.9	28–66	2–65	75.55	1.19
*ɣ-Proteobacteria*	*g_Luminiphilus (****11****), g_Litoricola (****3****), g_Nevskia (****1****), f_Methylophilaceae (****5****), f_Porticoccaceae (****1****), f_Pseudohongiellaceae (****5****), f_Shewanellaceae (****1****), o_Burkholderiales (****2****), o_SAR86 (****11****) o_Pseudomonadales (****5****), c_Gammaproteobacteria (****3****)*	51	1–4.1	31–69	1–86	70.34	1.29
*Bacteroidota*	*f_Cryomorphaceae (****4****), f_Flavobacteriaceae (****23****), o_Flavobacteriales (****8****), f_Balneolaceae (****2****), f_Crocinitomicaceae (****2****), c_Bacteroidia (****1****)*	44	1.19–2.57	28–58	4–43	73.91	0.94
*Thermoplasmatota*	*f_Poseidoniaceae (****6****), f_Thalassoarchaeaceae (****1****), g_Poseidonia (****6****)*	13	1.82–2.35	37–58	25–38	80.67	0.30
*Actinobacteriota*	*o_Nanopelagicales (****1****), g_Aquiluna (****1****), o_Actinomarinales (****5****), f_Ilumatobacteraceae (****4****), c_Thermoleophilia (****1****)*	13	1.23–2.22	32–71	2–23	76.79	1.45
*Cyanobacteria*	*g_Synechococcus_C (****10****)*	10	1.8–2.23	55–63	25–36	79.59	2.37
*Planctomycetota*	*f_Planctomycetaceae (****1****), o_Pirellulales; (****4****), p_Planctomycetota (****3****), g_Rubripirellula (****1****)*	9	3–6.4	49–72	45–132	87.14	0.95
*Verrucomicrobiota*	*o_Pedosphaerales (****2****), o_Opitutales (****1****), f_Puniceicoccaceae (****4****), f__Akkermansiaceae (****1****)*	8	2–4.8	42–60	23–74	83.51	4.21
*Marinisomatota*	*g_Marinisoma (****3****)*	3	0.8–0.93	31–32	2–3	55.31	0.36
*Margulisbacteria*	*c_ZB3 (****1****)*	1	1.71	42.5	10	67.53	0

Parenthesis () indicate the average value of each field in case of range values and number of MAGs in bold for each taxonomic affiliation. Taxonomic classification follows GTDB criteria. Marinisomatota includes former Marinimicrobia. Thermoplasmatota includes former Euryarchaeota group. d_:Domain, p_:Phylum, c_:Class, o_:Order, f_:family, g_:Genus, s_: Species. Av. (Average), Compl. (Completeness), Cont. (Contamination)

**Table 2:** Summary statistics and features of Black Sea 150 and 750 m retrieved MAGs

Phylum/Division	Taxonomic affiliation of MAGs (GTDB, Referenced groups)	***n***° of MAGs	Range Estimated Genome size (Mp)	Range GC (%)	Range median intergenic spacer (bp)	Av. Compl. (%)	Av. Cont. (%)
*Patescibacteria*	*c_Microgenomatia (****3****), c_Paceibacteria, c_ABY1 (****4****), o_Portnoybacterales (****2****), o_Shapirobacterales (****1****), o_Paceibacterales (****3****), o_Paceibacteria (****2****)*	15	0.6–1.69	30–41	17–62	58.09	1.24
*Omnitrophota*	*p_Omnitrophota;c_koll11 (****13****), f_Omnitrophaceae_A (****1****)*	15	0.84–3.78	35–47	7–84	70.45	1.69
*Planctomycetota*	*c_Brocadiae (****1****), c_Phycisphaerae (****7****), o_Pirellulales (****2****), o_Phycisphaerales (****1****), f_Pirellulaceae (****1****), p_Planctomycetota (****2****)*	14	1.92–14.18	43–71	22–110	72.33	1.78
*Desulfobacterota*	*o_Desulfatiglandales (****9****), o_Desulfobacterales (****4****), g_Desulfobacula (****1****)*	14	1.98–8.3	40–50	53–114	65.95	1.69
*Marinisomatota*	*o_Marinisomatales (****5****), c_Marinisomatia (****2****), p_Marinisomatota (****6****)*	13	1.6–4.4	34–44	10–62	81.18	1.20
*Chloroflexota*	*c_Anaerolineae (****3****), c_Dehalococcoidia (****3****), o_Anaerolineales (****4****), o_Dehalococcoidales (****3****)*	13	1.45–5.78	48–63	32–89	71.59	2.14
*α-Proteobacteria*	*o_Rhodospirillales_A (****6****), f_Magnetospiraceae (****1****), c_Alphaproteobacteria (****4****)*	11	2.59–4.72	53–64	24–62	73.51	0.98
*Nanoarchaeota*	*o_Woesearchaeales (****5****), o_Pacearchaeales (****1****), c_Nanoarchaeia (****3****)*	9	0.8–1.6	28–36	23–70	64.72	1.50
*Bacteroidota*	*s_Chlorobium_A phaeobacteroides (****1****), c_Ignavibacteria (****1****), o_Bacteroidales (****5****), c_Bacteroidia (****1****)*	8	2.46–5 (3.5)	33–49	40–81	85.09	2.28
*ɣ-Proteobacteria*	*g_Thioglobus_A (****2****), g_Methylobacter_A (****1****),g_Acidovorax_D (****1****), c_Gammaproteobacteria (****3****)*	7	1.18–5.59	37–64	14–67	75.25	2.11
*Crenarchaeota*	*c_Bathyarchaeia (****3****), g_Nitrosopumilus (****3****)*	6	1.39–2.92	32–58	37–85	65.18	0.88
*Actinobacteriota*	*o_Microtrichales;f_MedAcidi-G1 (****2****), p_Actinobateriota;c_UBA1414 (****2****)*	4	1.42–2.33	31–64	27–68	76.65	1.09
*Verrucomicrobiota*	*c_Kiritimatiellae (****2****), o_Kiritimatiellales (****2****)*	4	2–3.6	53–60	45–56	74.04	2.53
*Aenigmarchaeota*	*c_Aenigmarchaeia (****3****), o_Aenigmarchaeales (****1****)*	4	0.71–1.29	36–45	37–59	55.9	1.86
*Cloacimonadota*	*c_Cloacimonadia (****2****), o_Cloacimonadales (****1****)*	3	1.2–2.91	31–36	15–50	74.34	0.11
*Campylobacterota*	*f_Arcobacteraceae (****1****), g_Sulfurimonas (****1****)*	2	1.14–1.81	30–35	9–15	60.01	1.04
*Nitrospirota*	*c_Thermodesulfovibrionia (****2****)*	2	1.85–3.18	44–46	53–68	68.81	0.94
*KSB1*	*p_AABM5–125-24 (****1****), p_KSB1 (****1****)*	2	4.05–5.13	37–46	94–144	73.31	2.2
*Myxococcota*	*p_Myxococcota (****1****)*	1	4.46	63.7	37	60.65	0.84
*Bdellovibrionota*	*f_Bacteriovoracaceae (****1****)*	1	4.78	37.2	40	92.41	3.63
*Spirochaetota*	*c_Spirochaetia (****1****)*	1	2.92	40.9	52	67.32	1.89
*Halobacterota*	*d_Archaea;p_Halobacterota;c_Syntrophoarchaeia;o_ANME-1 (****1****)*	1	1.81	43.1	52	55.52	0.65
*Nitrospinota*	*f_Nitrospinaceae (****1****)*	1	3.08	46	69	93.96	2.56
*Delongbacteria*	*p_Delongbacteria (****1****)*	1	3.002	54	45	64.03	1.1
*SAR324*	*c_SAR324;o_SAR324 (****1****)*	1	2.96	41.7	53	82.86	0
*SM23*	*d_Bacteria;p_AABM5–125-24 (****1****)*	1	3.58	45.1	131	73.63	3.3
*Acidobacteriota*	*f_Aminicenantaceae (****1****)*	1	3.0955127	40.5	68	89.52	4.27

Parenthesis () indicate the average value of each field in case of range values and number of MAGs in bold for each taxonomic affiliation. Taxonomic classification follows GTDB criteria. Marinisomatota includes former Marinimicrobia. Halobacterota includes former Euryarchaeota methanogens group. Campylobacterota includes former Epsilonproteobacteria. d_:Domain, p_:Phylum, c_:Class, o_:Order, f_:family, g_:Genus, s_: Species. Av. (Average), Compl. (Completeness), Cont. (Contamination)

**Fig. 4:**
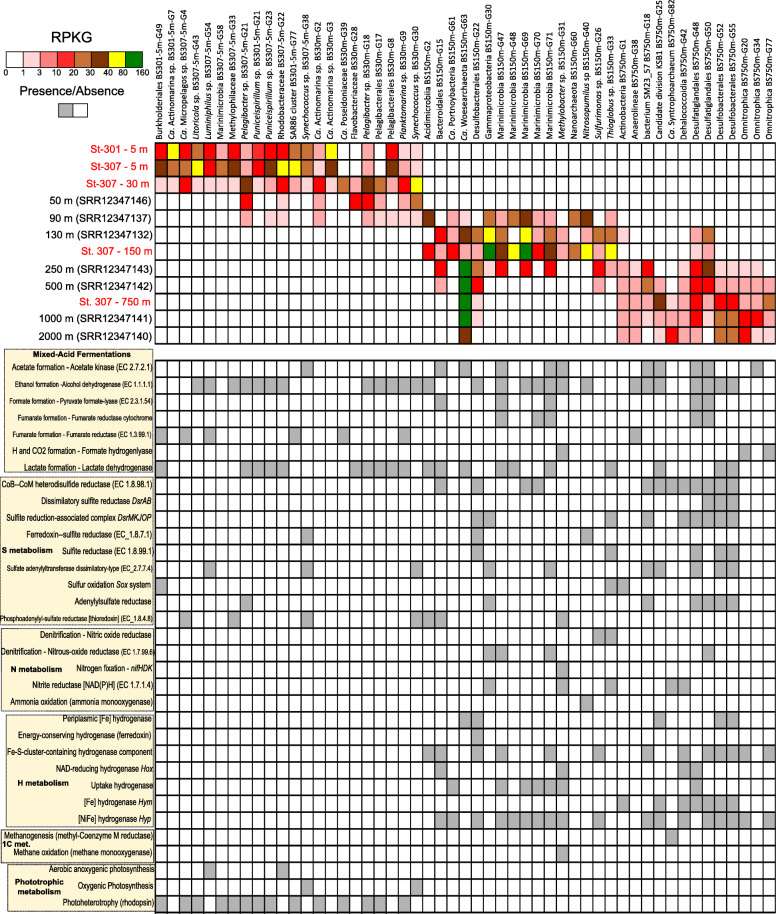
Recruitment analysis of the 50 most abundant Black Sea MAGs retrieved from our datasets (in red) and detected at highest values at various Black Sea metagenomes from the NCBI (Bioproject PRJNA649215). Reads were recruited at > 95% of identity and > 50 bp of alignment lengths. The predominant metabolism is characteristic of each MAG and was detected in the genome with KEGG, SEED, COG, TIGFRAMs and NCBI-nr databases

Among all of the MAGs retrieved from this work, we selected the 50 most abundant MAGs (> 10 RPKGs in any of the recruited samples) from the oxic, redoxcline, and anoxic waters and recruited them at > 95% of identity (species level) on all metagenomes and showed their main distinguishing metabolic features (Fig. 4). The rest of the MAGs abundance in all datasets is shown in detail in Additional File 3.

### MAGs from the epipelagic and DCM oxic strata

As expected, emblematic key players of the oxic waters harboring a photoheterotrophic/phototrophic lifestyle such as Ca. Pelagibacter, Ca. Actinomarina, SAR86, *Synechococcus,* or Flavobacteriaceae were detected in high numbers in all oxic metagenomic datasets. As noted above, we must highlight the complete absence of *Prochlorococcus* spp. genomic footprints (with no significant genome recruitment above 70% of identity with any existing *Prochlorococcus* genome), contrasting with a high abundance of *Synechococcus* MAGs that affiliated with the marine clades I, III, IV, VI and WPC1 including isolates (KORDI-49, BL107, CC9902, WH 8016, WH 7805/7803, WH 8103/8102) [18]. The main Actinobacteria MAGs retrieved presented relatively small genome sizes (1.2–2.2 Mb), among which we must highlight the presence of 5 novel *Actinomarinales* spp. (BS301–5 m-G7**,** BS307–5 m-G2, BS30m-G2/G3/G4) and a group of *Ilumatobacteraceae* genomes related to Caspian MAGs (Casp-actino5) [13]. The major SAR11 Alphaproteobacterial MAGs were eight novel *Pelagibacterales* that affiliated with the recently described groups Ia.1, IIaB/1and IIIa [19]. Remarkably, we obtained three novel MAGs from the order *Rickettsiales*, that showed similarities with groundwater aquifer MAGs (< 80% of ANI) and freshwaters from Lake Baikal [17]. Another relevant Alphaproteobacteria clade from which we obtained MAGs was SAR116, with six MAGs affiliated to the *Puniceispirillum* genus and five more were only classified as representatives of the family Puniceispirillaceae. A family that provided abundant MAGs in the Black Sea oxic waters is Flavobacteriaceae (23 MAGs), a group that was commonly detected in the Mediterranean [12] and Baltic Seas [20]. Various MAGs were related at GTDB genus level with MED-G11, MED-G14 MAGs, and at the species level (ANI > 95%) with MED-G20 Mediterranean Sea MAGs. Two MAGs also showed their closest relatives at the GTDB family level with Baltic Sea MAGs BACL11 and at the species level with BACL21. We also found five methylotrophic representatives from the clade OM43 (family *Methylophilaceae*) affiliating at the genus level to BACL14 Baltic Sea MAGs [20]. Eleven MAGs belonged to the cosmopolitan Gammaproteobacteria SAR86, so far only classified at this order level. Other Gammaproteobacteria  that co-occurred in these samples were MAGs with similarity to *Luminiphilus* (11 MAGs) [21] and *Litoricola* (3 MAGs) [22] genera. Another relevant taxon from marine systems was the former marine group-II Euryarchaeota (Thermoplasmatota according to GTDB taxonomy). We retrieved six genomes affiliating to the family *Poseidoniaceae* and the other six to the genus *Poseidonia*. Only one genome was obtained affiliating to Thalassoarchaeaceae. Finally, three ultra-small (1 Mb of estimated genome size) Marinimicrobia MAGs were obtained from the oxic metagenomes, which so far are classified by the GTDB as genus *Marinisoma*.

### MAGS from the redoxcline

Next, we showed the main ecological drivers of the redoxcline layer, which included chemolithotrophic S oxidizers and C fixers such as Ca. Thioglobus and dissimilatory nitrate reducers such as *Sulfurimonas*, both of which were recently described as members of the Black Sea microbiome [9]. The redoxcline also showed some other ecologically relevant nitrate reducers such as Bacteroidales MAGs, ammonia oxidizers such as *Nitrosopumilus* spp., sulfate reducers *Desulfatiglandales* and *Desulfococcales* novel species, and several *Marinimicrobia* representatives which are specialized in the H metabolism, denitrification, and mixed-acid fermentation. We noticed the presence of anoxygenic photosynthesis, exemplified by MAG BS150m-G13 showing > 99% of ANI with *Chlorobium phaeobacteroides*, a green sulfur bacterium (GSB) originally isolated from the Black Sea [23] (GCA_000020545.1), that was undergoing a nearly monoclonal bloom (Fig. S4). Chemoautotrophy was detected in *Thioglobus* sp. BS150m-G29 and G33 MAGs, both of which are novel representatives of the SUP05 clade which perform a wide variety of metabolisms including S oxidation and C fixation, and with only 80% of ANI with its closest relative (Ca. Thioglobus autotrophicus EF1) [24]. It appears that this is a case of a single species (recruiting at > 95% of nucleotide identity) abundant (> 70 RPKG, Fig. S5) in the Black Sea redoxcline. Methane oxidation (*Methylobacter* sp. BS150m-G31) and ammonia oxidation were also metabolisms observed in MAGs from this layer (*Nitrosopumilus* spp. BS150m-G38/39/40). Nitrite oxidation was detected in *Nitrospinaceae* BS150m-G45.

Denitrification was frequently detected among redoxcline MAGs, although complete denitrification including the last step involving conversion of nitrous oxide into nitrogen gas (*nosZ* gene) was seen only in five MAGs (*Marinimicrobia* BS150m-G46/G47/G71, unclassified *Alphaproteobacteria* BS150m-G7/G9, *Rhodospirillales* BS150m-G4/G10, *Sulfurimonas* sp. BS150m-G26 and unclassified *Gammaproteobacteria* BS150m-G28/30/32). Dissimilatory nitrate reduction to ammonium (*nrfAH* genes) was far more restricted and found in *Marinimicrobia* BS150m-G46, *Campylobacterota* (*Sulfurimonas* sp. BS150m-G26), or *Bacteroidales* BS150m-G15. Nitrate reduction through the *nirB* gene was much more widely detected including in all *Alphaproteobacteria* MAGs and various *Gammaproteobacteria* members. N fixation (nifDK dinitrogenase subunits) was detected in only two MAGs (*Chlorobium phaeobacteroides* and *Nitrospirota*, BS150m-G55/G56 respectively).

Dissimilatory sulfate reduction and oxidation (*dsrAB* genes) showed up already in this sample in various genomes such as *Desulfobacterota, Nitrospirota* MAGs, *Planctomycetota* (*Pirellulaceae* BS150m-G36) (already mentioned above), *Chloroflexota* (*Anaerolineales* BS150m-G18), *Alphaproteobacteria* (*Rhodospirillales* BS150m-G3/G4/10/G11), *Chlorobium phaeobacteroides* MAG and *Gammaproteobacteria* (Ca. Thioglobus and *Gammaproteobacteria* BS150m-G28 MAGs). It must be noted that, among the main features of this habitat, there was the simultaneous activity of sulfate-reducing and sulfide-oxidizing microbes forming part of the same ecological niche, a process known as cryptic sulfur cycle [25]. However, low O_2_ concentrations (0.87 mg/L) and low ratio (0.16) of catalase and peroxidase/recA genes (1.5 in oxic datasets) demonstrate the microaerophilic/anoxic nature of this habitat.

We also assessed the presence of our redoxcline MAGs with previously available metagenomes from the redoxcline from the Cariaco Basin (Venezuela) [10]. Overall, it seems that *Marinisomatota/Marinimicrobia* and *Gammaproteobacteria* chemolithotrophic groups are the most abundant key players of these two marine redoxclines, accounting for more than 50% of total microbial biomass (Fig. S2A). However, it must be noted that only a few MAGs from the Black Sea were detected there (Fig. S2C). Among them, two chemolithotrophic Gammaproteobacterial representatives (Ca. Thioglobus and a novel species BS150m-G30 classified only at the order level as o__GCA-2,400,775 by GTDB), sulfate reducers (*Desulfatiglandales*), denitrifying and hydrogen-producing *Marinimicrobia* (three species) and one *Actinobacteriota* (a novel species from the marine MedAcidi-G1 group). Apart from their metabolic potential fitting with microbial lifestyles from redoxcline layers, these species could play key roles in other marine redoxclines and oxygen minimum zones (OMZs), as their detection in two largely separated biomes with different salinities (ca. 2% in the Black Sea and 3.5% in the Cariaco Basin) indicate a widespread distribution in oxygen-depleted marine niches.

### Euxinic MAGs and the “microbial dark matter”

The main environmental variables grouping with the mesopelagic sample of 750 m were PO_4_ and Si, both of which are solubilized in anoxic layers and diffuse from the sediment layer (Figs. 2 and 3). Salinity also increased up to 2.2% in these euxinic waters. There is the expected predominance of sulfate reduction pathways, as carried out by *Desulfobacterota* MAG representatives (*Desulfatiglandales* BS750m-G47-G51 and BS750m-G54/G56, *Desulfobacterales* BS750m-G52/G53/G55), which perform the dissimilatory sulfate reduction pathway (*dsr* genes). MAGs that were detected at all euxinic strata included various other sulfate-reducers, their associated microbiota performing mixed-acid fermentations and H metabolism in syntrophism (*Cloacimonadetes*, *Woesearchaeales*, Ca. Aminicenantes) and the only MAG able to perform methanogenesis and ANME, a *Syntrophoarchaeum* that showed a remarkable abundance in 1000 and 2000 m metagenomic datasets, suggesting that methane metabolism indeed coexists with sulfate reduction and associated microbial fermenters and H_2_ scavengers in a complex syntrophic network.

The key methanogenesis genes (*mcr* and *fwd*) were detectable in the abovementioned single MAG, Ca. Syntrophoarchaeum BS750m-G82. However, other genomes classified as *Bathyarchaeota* BS750m-G27/G28 showed most of the methanogenesis genes except for the key enzyme, Methyl-coenzyme M reductase (*mcr*). These enigmatic *Bathyarchaeota* representatives showed various mixed-acid fermentation pathways including the formation of H_2_ and CO_2_ (via the formate hydrogenlyase), formate (pyruvate-formate lyase), alcohol (alcohol dehydrogenase), or lactate (lactate dehydrogenase). It must be noted the potential capability of performing reverse methanogenesis, or anaerobic methane oxidation (ANME) by the abovementioned archaeon (MAG Ca. Syntrophoarchaeum BS750-G82). Heterodisulfide reductase genes (*HdrABC*), which are involved in electron transfer and the last step of methanogenesis by reducing CoB-CoM heterodisulfide, were detected in *Bathyarchaeota* and *Syntrophoarchaeum* MAGs. However, these genes were also found in *Cloacimonadota*, candidate division KSB1, Ca. Aminicenantes, *Omnitrophota*, *Desulfobacterota*, *Planctomycetes*, and *Chloroflexi* MAGs as well as in the unassembled reads (being completely absent from oxic datasets), suggesting that these electron transfer complexes are not exclusive to methanogens. As seen by the dbRDA, we also noted a global predominance of mixed-acid fermentation pathways (with ethanol, lactate, acetate, formate, or CO_2_/H_2_ as products) and hydrogen uptake hydrogenases that couple with sulfate, fumarate, CO_2,_ or nitrate reduction, thus conforming a complex syntrophic network of microbes. This networking of syntrophic microbes (considered here as interspecies H transfer) includes the abovementioned uncultured taxa plus accompanying streamlined members of the “microbial dark matter” such as *Omnitrophota*, Patescibacteria (Ca. Microgenomates, Portnoybacteria, Paceibacteria) or Nanoarchaeota (Ca. Aenigmarchaeota, Woesearchaeota, Pacearchaeota), groups from which we also obtained MAGs (see Table 2). Various types of hydrogenases and hydrogen metabolism pathways grouped with the 750 m mesopelagic sample in the dbRDA plot (Fig. 2) and were found in the vast majority of microbes inhabiting this sulfide enriched waters, including NAD-reducing bidirectional (*hox* genes) and uptake hydrogenases (*hup* genes), NiFe (*hyp* genes) and FeFe (*hym* genes) hydrogenases, Coenzyme F420-reducing hydrogenases or carbon monoxide induced hydrogenases (*CooHL* genes), all of which showed the highest gene/ recA ratios (from 0.2 in *hym* genes to 1–2 for *hyp* and *hoxF*) in euxinic waters.

It was remarkable the presence of two *Actinobacteriota* MAGs (BS750m-G1/G2) in these sulfide-rich waters. These yet unclassified members have their highest resemblance with MAGs retrieved from groundwater aquifers (*Actinobacteriota* bacterium CG08_land_8_20_14_0_20_35_9, classified as UBA1414 by GTDB) and have very low GC content (31–34%) and predicted genome sizes (ca. 1.4–1.6 Mb). Their genomes presented various mixed-acid fermentative pathways associated with the production of ethanol (alcohol dehydrogenase), lactate (lactate dehydrogenase), formate (pyruvate-formate lyase), and H/CO_2_ (formate hydrogen lyase). They also showed an active hydrogen metabolism with various NiFe hydrogenases including Coenzyme F420-reducing hydrogenase, *hyp* genes, and HyaA COG1740 355 Ni-Fe-hydrogenase I. Another remarkable group of microbes was *Omnitrophota*, from which we obtained 15 MAGs with variable estimated genomes sizes (from 1 to 3 Mb). For instance, the most abundant *Omnitrophota* MAG retrieved from our samples (BS750m-G77) presented a small predicted genome size (ca. 1.2 Mb) and was an obligate fermenter (mainly producing ethanol, H_2_/CO_2,_ and lactate). Another group of streamlined members of the microbial dark matter was *Aenigmarchaeota* (BS750m-G24/36/81/83/) and *Nanoarchaeota* (BS750m-G11/13/70) MAGs, which had estimated genome sizes of 1–1.5 Mb. Among their metabolic potentials, they were also mixed-acid fermenters, including lactate or H_2_/CO_2_ as fermentation by-products, which would fuel the sulfate reducers, conforming a syntrophic network with the rest of the mixed-acid fermenters. Finally, another set of microbes of small genome sizes (0.6–1.6 Mb) were *Patescibacteria* (former Candidate Phyla Radiation). We must highlight the presence of the protein VirB4, associated with type IV secretion systems that work as injectors into host cells [26], in Ca. Microgenomates BS750m-G73/74, Ca. Paceibacteria BS750m-G71/75 and Ca. Portnoybacteria bacterium BS750m-G76. These proteins were only found in these microbes in the entire euxinic waters, which suggests a parasitic lifestyle in which these *Patescibacteria* could translocate nutrients, proteins, and DNA from or to a putative host [26].

## Discussion

The first ecogenomic insights of Black Sea water column microbiota showed, at first sight, that this brackish microbiome has various taxa previously observed in other brackish systems such as Caspian [13] or Baltic Seas [20]. However, its interconnection with the Mediterranean Sea has allowed the coexistence of some other microbes not found in the other two [12]. However, the uniqueness of this enormous brackish water mass lies in its redoxcline and very deep euxinic strata, features that are not present in the Caspian or Mediterranean Seas.

The present study, together with others from the Black Sea [5, 9] (bioproject PRJNA649215) and the Cariaco Basin in Venezuela [10, 11] provide a first glimpse into the microbiome of anoxic marine water columns and Oxygen Minimum Zones (OMZs). The redoxclines of these habitats show a convergence of various coexisting metabolisms, among which we encounter anoxygenic photosynthesis such as that one observed in *Chlorobium phaeobacteroides* [27], ammonia oxidation by *Nitrosopumilus* spp., chemolithotrophic metabolisms carried by *Gammaproteobacteria* such Ca. Thioglobus, one of the most abundant and versatile players transitioning between oxic-anoxic regimes [24, 28]. This microbe has been detected both in Cariaco and Black Sea basins [9] and its adaptive metabolism, which includes physiological adaptations to oxic-anoxic growth [28] and its broad metabolic potential has led it to colonize these zones with a large contribution to S and N biogeochemical cycles as a denitrifier, sulfur-oxidizing, and C fixer chemolithotroph. The simultaneous activity of sulfate-reducing and sulfide-oxidizing microbes in these habitats has led to a term known as the “cryptic sulfur cycle” [25]. One of the most abundant microbes detected from these zones belongs to the *Marinimicrobia*, which are specialists of both oxic and anoxic waters [29, 30], able to perform denitrification and various fermentations and H_2_ metabolism and were recently labeled as organoheterotrophs with specific molybdoenzymes to generate energy from sulfur cycle intermediates [9].

As we approach the euxinic meso and bathypelagic waters, we encountered domination of sulfate reduction coupled with a complex variety of syntrophic networks that feed the ecosystem. In this sense, *Desulfobacterota* is a good example of a syntrophic phylum that could be able to accept electrons from organic electron donors, as noted previously in marine sediments [31]. It appears that the extremely high abundance of sulfate-reducers in the Black Sea has displaced methanogens, which are present in the water column but low in numbers. We found MAGs of Bathyarchaeota [32, 33] representatives and a single example of a *Syntrophoarchaeum* in this study. This microbe could be performing reverse methanogenesis or anaerobic methane oxidation (ANME) rather than the standard methanogenesis. Recently, members of this newly identified species performed the complete oxidation of butane during the anaerobic methane oxidation process [34]. However, it appears that the competition between methanogens and sulfate-reducers for acetate is dominated by the latter, which also take a fundamental role in the syntrophic networks by utilizing the H_2_ produced by fermentation. Among all of the associated microbiota, we must highlight the presence of the *Cloacimonadota* phylum (previously known as WWE1), which have been detected in meromictic lakes as important carbon and sulfur recyclers [35] and appear to degrade propionic acid in syntrophic networks in bioreactors [36]. Novel microbes from lineages such as *Cloacimonadota* (WWE1), *Marinisomatota* (SAR406), *Omnitrophicaeota* (OP3), *Bacteroidales, Kiritimatiellae, Anaerolinea/Dehalococcoidia* formed a very complex syntrophic network where mainly mixed-acid fermentations with lactate, ethanol, formate, succinate, hydrogen, and CO_2_ are the final products. Other microbes obtained as MAGs included members of the uncultured microbial dark matter, such as *Patescibacteria* and *Nanoarchaeota* [6, 37–39]. In particular, we have stumbled upon various members of *Aenigmarchaeota* [40] and *Woesearchaeales* with their usual streamlined genomes and hard to predict metabolic properties.

The study of microbes from this and other brackish water bodies could be useful in understanding better the reasons for the great divide between fresh and marine water bodies and shed light upon the physiological and genomic strategies that are responsible for marine- freshwater microbial transitions, considered infrequent [41] and demanding in terms of physiology and protein/gene variation [42], that have been detected in several groups of aquatic microbes.

## Methods

### Sampling, DNA extraction, physical and chemical profiles measurement

Samples for metagenome analyses were collected from St. 301 (5 m deep) and St. 307 (5, 30, 150, and 750 m deep) in October 2019 (coordinates 43.155517 N 28.005383 E and 43.1696 N and 29.001283 E, respectively). Up to 6.9 L of seawater from each sampling depth were filtered through a series of 20 μm Nylon Net filters (Millipore), 5 μm polycarbonate membrane filters (Millipore), and 0.22 μm SterivexTM Filter Units (Merck). DNA was then extracted only from these Sterivex filters using standard phenol-chloroform protocol [43]. In short, Sterivex filters were treated with CTAB lysis buffer and then treated with 1 mg ml^− 1^ lysozyme and 0.2 mg ml^− 1^ proteinase K (final concentrations). Then nucleic acids were purified with phenol/chloroform/isoamyl alcohol and chloroform/isoamyl alcohol.

### Sequencing, assembly, and read annotation

The five samples were sequenced in one lane of Illumina HiSeq X 10 PE 2X150 bp (Novogene company), which provided ca. 180–200 million clean reads and 24 Gb of output for each sample. Samples were individually assembled using IDBA-UD [44] with the parameters --pre_correction, −-mink 50, −-maxk 140, −-step 10.Sub-assemblies of 20 million reads were done in each sample to retrieve some of the most abundant microbes that assembled poorly i.e. we reduced the total number of reads to obtain more complete bins of these representatives (e.g. *Pelagibacterales*, *Actinomarinales*, *Synechococcus*, *Marinimicrobia* or *Thioglobus* spp.). Annotation of contigs was assessed using Prodigal [45] for ORF prediction and then BLAST (nr database) using Diamond for functional annotation [46]. Proteins were annotated with customized nr, COG [47], and TIGFRAM [48] databases (last releases from January 2020) that served as a taxonomic proxy to understand what we obtained from our datasets. Features like tRNAs and rRNAs were detected with tRNAscan [49] and ssu-align [50], respectively.

### Binning, classification, and MAG statistics

The Binning procedure was performed as follows: a first manual inspection was done assigning a hit (based on BLAST against Nr) to each CDS (coding sequence), which allowed us to classify contigs taxonomically into different phyla. This step, although not necessary for current binning approaches, has provided us good results in past studies of emblematic aquatic systems such as the Mediterranean Sea or the Lake Baikal [12, 51]. Then, an initial binning step was applied for each set of contigs assigned to each phylum with METABAT2 [52] using coverage in the different samples. When METABAT2 provided a single bin containing various species, we applied a manual inspection of the annotated contigs and we used other computational approaches based on, GC content, RPKGs abundance values, and tetranucleotide frequencies to refine and separate the bins [53, 54]. Finally we only used MAGs with < 5% contamination and > 50% of completeness based on CheckM estimations [55]. MAGs were taxonomically classified according to the latest version of GTDB-tk and the database release89 [16] and whenever we could we used class, order, family, genus, or species names for all of them.

### 16S rRNA gene read classification, hierarchical cluster read analysis, read functionality

The 16S rRNA gene reads were detected in a subset of 20 unassembled million reads from each metagenome. We first obtained candidates using USEARCH [56] with RefSeq 16S rRNA gene as database and then these putative 16S rRNA genes were confirmed using ssu-align [50]. Then, a BLASTN was performed against the SILVA database [14] (SILVA_138_SSURef_Nr99_Tax_silva from December 2019) to provide a taxonomic classification. Hierarchical cluster analysis (dendrograms) of different metagenomic samples with k-mer = 21 bp was assessed with SIMKA [57] and Bray-Curtis indexes of presence/absence were obtained. Subsets of 20 million reads of each metagenomes were analyzed with BLASTX against SEED [15] database using Diamond [46], with parameters more-sensitive, max-target-seqs 1, e-value 0.00001 > 50 bp of alignment length and > 50% identity. The top hits were analyzed in search of specific genes and pathways based on the SEED database. Hits were normalized by the total number of hit counts for each sample and a row Z-score was calculated to assess statistical differences between samples for each metabolic pathway.

### Relative abundance of MAGs

To estimate the relative abundance of the recovered genomes in various datasets we performed read recruitment and mapping, which was assessed considering BLASTN hits of the metagenomic reads against each MAG at > 95% identity and 50 bp of alignment length thresholds, as indication of belonging to the same species. A microbe was considered present in a metagenome if it was detected at > 1 RPKG (Reads per Kb of Genome per Gb of Metagenome). All relative abundances of our MAGs on Black Sea datasets are shown in Additional File 3. Datasets used for recruitment included Black Sea (PRJNA649215) and Cariaco Basin (PRJNA326482).

### Redundancy analysis (RDA) of environmental variables, MAGs, and metabolic processes

Distance-based redundancy analysis (dbRDA) analysis was performed to describe the ordinations of the main MAGs and metabolic processes in an environmentally constrained space [58] and conducted with the R package vegan [59]. Environmental matrixes were constructed with 12 environmental variables for the 5 Black Sea samples. Each matrix was square-root transformed and normalized and subsequently transformed to Euclidean resemblance matrix. On the other hand, we constructed the other two matrixes with the recovered MAGs and metabolic processes with 30 selected metabolic processes. Both were standardized and square root transformed before performing a Bray–Curtis dissimilarity resemblance matrix. Two dbRDA were obtained, the first one comprising the recovered genomes (MAGs) matrix using environmental matrix as predictor variable, and a second one based on the metabolic processes matrix using environmental matrix as predictor variable.

### Availability of data and materials

All metagenomes and reconstructed genomes derived from this work are publicly available under the NCBI Bioproject PRJNA638805. Metagenomes were deposited to NCBI-SRA with the accession numbers SRR12042682-SRR12042686.

## Supplementary Information

**Additional file 1: Table S1.** Main features and metadata of different Black Sea stations from which metagenomes were obtained.

**Additional file 2: Table S2.** Main features and statistics of Black Sea retrieved MAGs.

**Additional file 3: Table S3.** Reads per Kb of Genome per Gb of metagenome (RPKGs) of all Black Sea MAGs on different Black Sea metagenomes.

**Additional file 4: Figure S1.** Comparison of Black Sea metagenomic datasets from the present study (in red) and those available from the NCBI database (Bioproject PRJNA649215). Comparison made at the level of A) Phylum 16S rRNA gene taxonomic classification, B) Heatmap read cluster analysis with Bray-Curtis presence/absence indexes.

**Additional file 5: Figure S2.** Comparison of Black Sea and Cariaco Basin redoxclines at the level of A) 16S rRNA gene taxonomic classification, B) Hierarchical read cluster analysis with Bray-Curtis presence/absence indexes. C) Black Sea MAG species recruiting at the Cariaco depth profile datasets (PRJNA326482). Recruitment was assessed with > 95% of identity and > 50 bp of alignment lengths.

**Additional file 6: Figure S3.** Estimated genome size (Mb) versus GC content of all Black Sea MAGs retrieved in this work. Shape indicates the depth at which the MAG was recovered. MAGs are color-coded at the phylum level.

**Additional file 7: Figure S4.** Recruitment plot of *Chlorobium phaeobacteroides* BS150m-G13 from the Black Sea 150 m redoxcline metagenome. Each dot represents a mapped read at > 95% of identity and > 50 bp of alignment lengths.

**Additional file 8: Figure S5.** Recruitment plot of *Thioglobus* sp. BS150m-G33/G29 from the Black Sea 150 m redoxcline metagenome. Each dot represents a mapped read at > 95% of identity and > 50 bp of alignment lengths.

## Data Availability

All data derived from this work is publicly available in the NCBI-Genbank databases.
