# Supplementary figures and images for: The microbiome of the Black Sea water column analyzed by shotgun and genome centric metagenomics
Source: Environ Microbiome. 2021 Mar 16;16:5. doi: 10.1186/s40793-021-00374-1 (PMC8067304; doi:10.1186/s40793-021-00374-1)

A

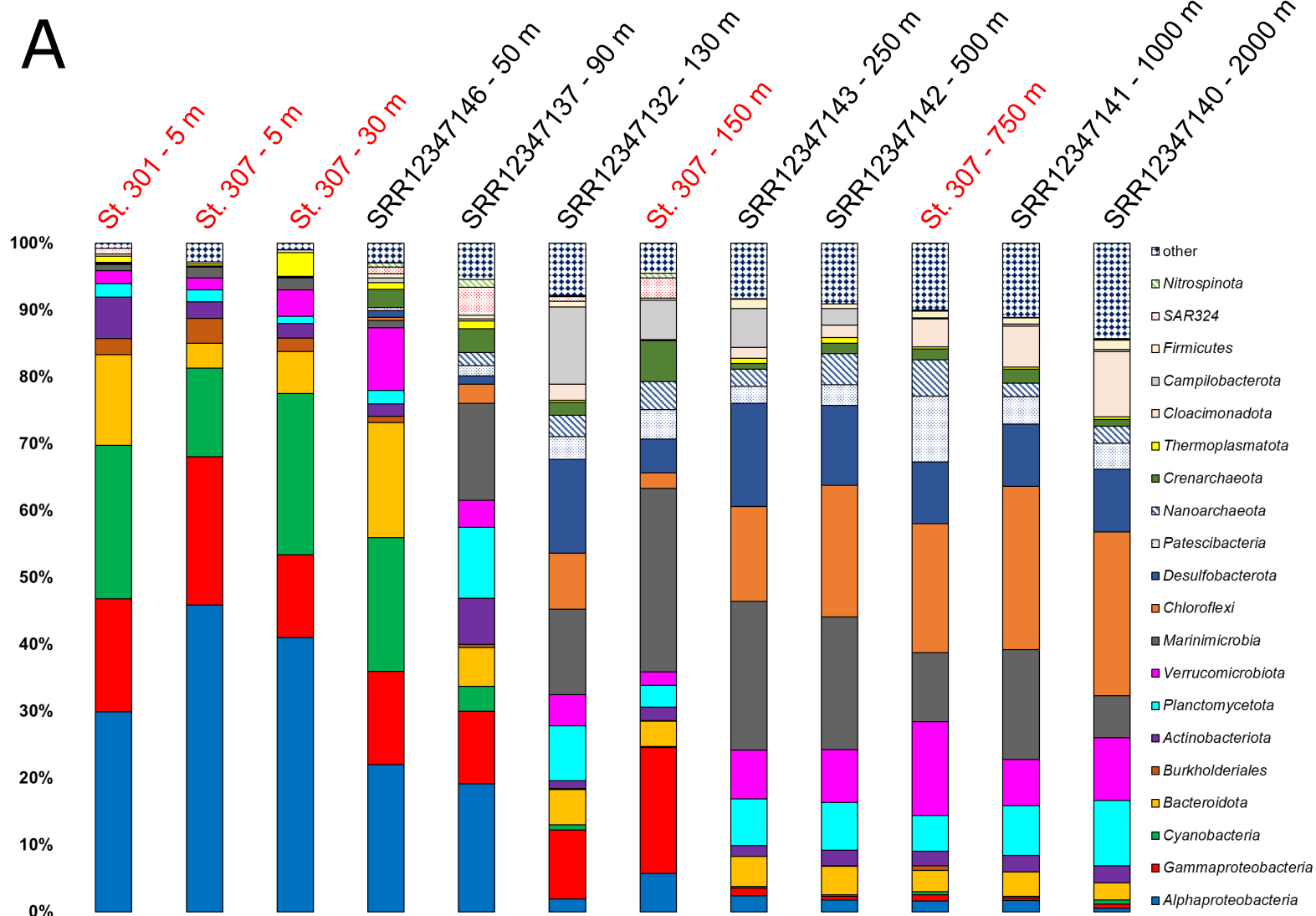

B

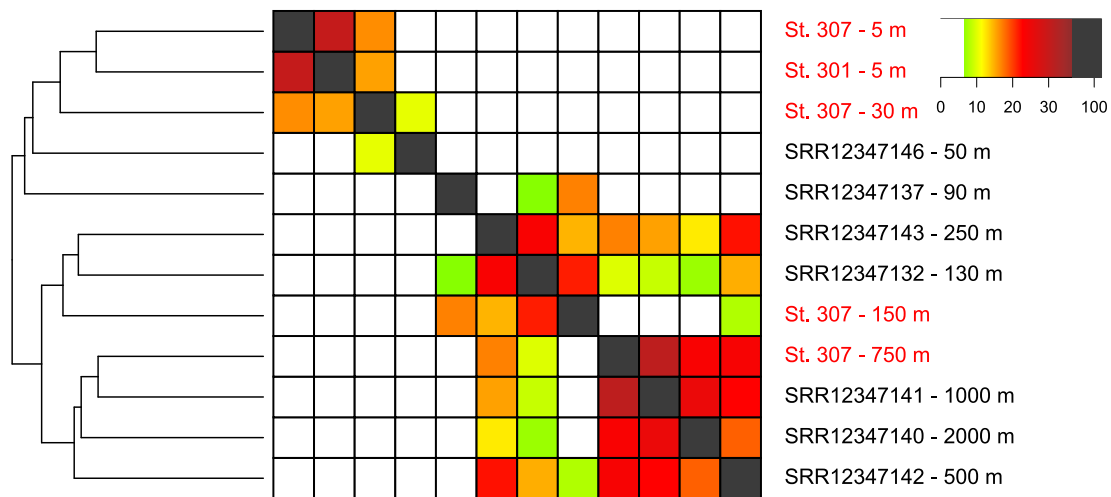

Supplement: Supplementary file 4 — Additional file 4: Figure S1. Comparison of Black Sea metagenomic datasets from the present study (in red) and those available from the NCBI database (Bioproject PRJNA649215). Comparison made at the level of A) Phylum 16S rRNA gene taxonomic classification, B) Heatmap read cluster analysis with Bray-Curtis presence/absence indexes. [file 40793_2021_374_MOESM4_ESM.pdf]

A

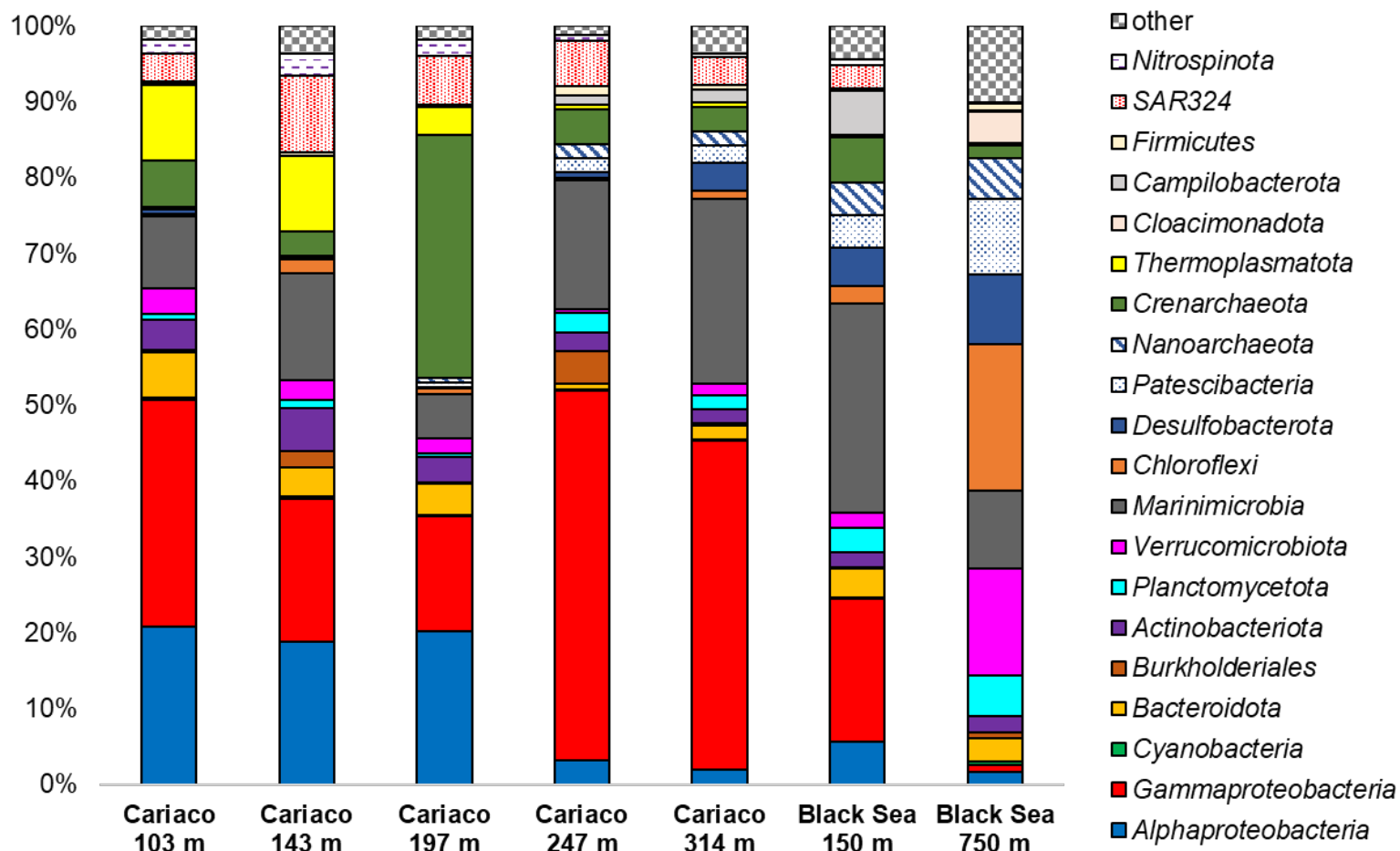

B

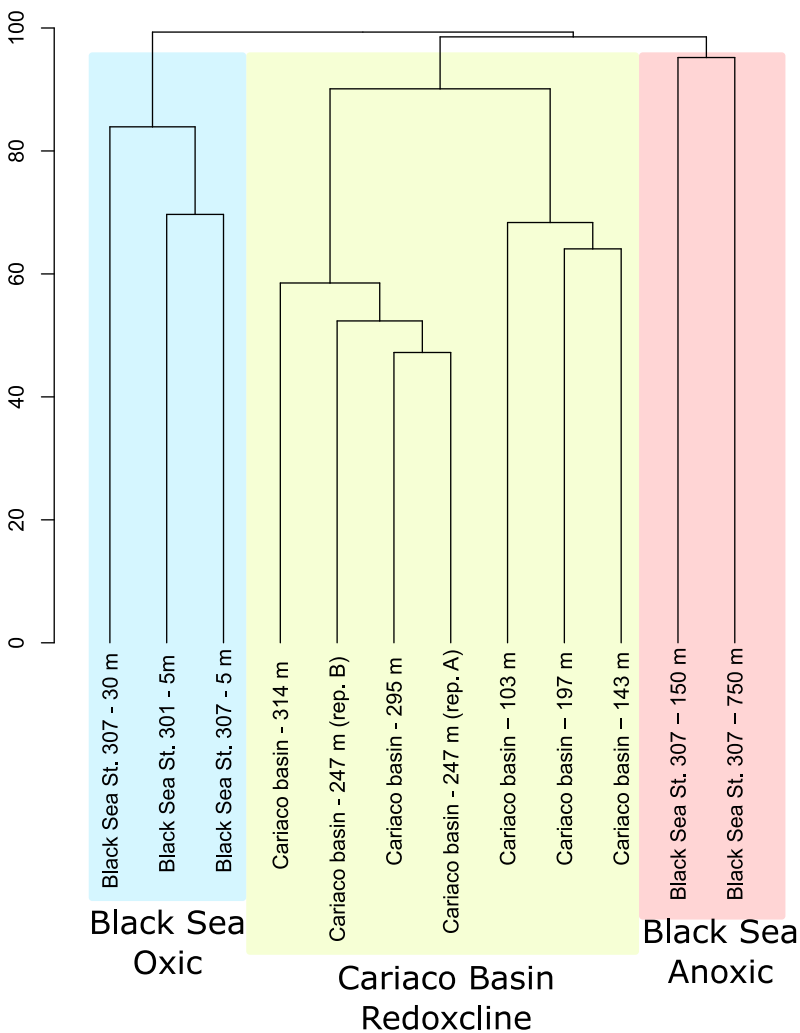

C

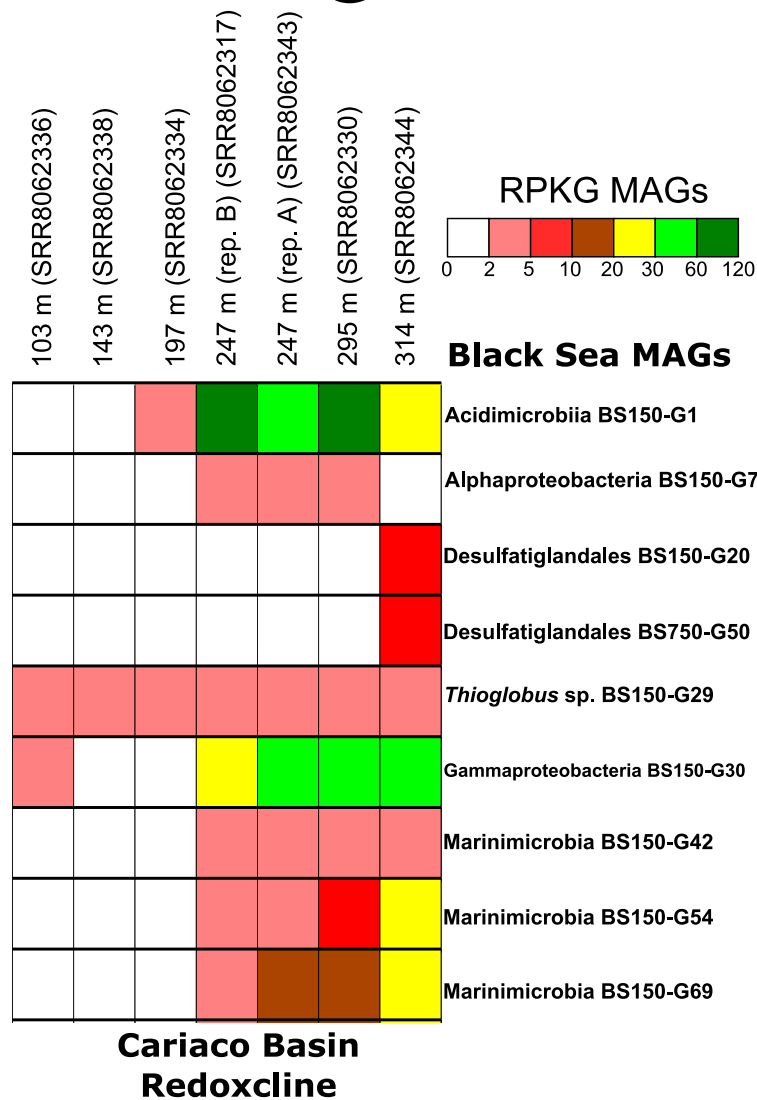

Supplement: Supplementary file 5 — Additional file 5: Figure S2. Comparison of Black Sea and Cariaco Basin redoxclines at the level of A) 16S rRNA gene taxonomic classification, B) Hierarchical read cluster analysis with Bray-Curtis presence/absence indexes. C) Black Sea MAG species recruiting at the Cariaco depth profile datasets (PRJNA326482). Recruitment was assessed with > 95% of identity and > 50 bp of alignment lengths. [file 40793_2021_374_MOESM5_ESM.pdf]

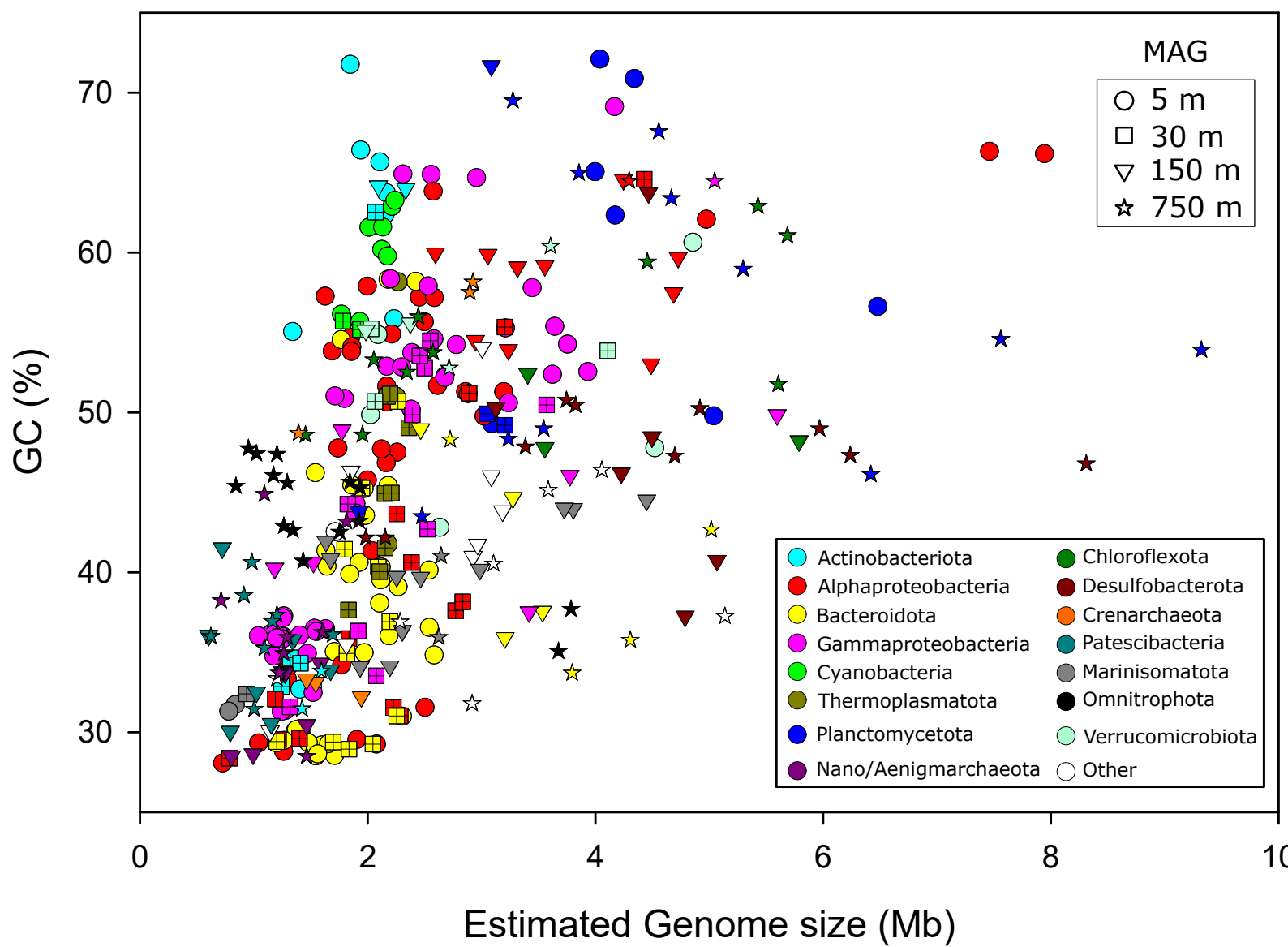

Supplement: Supplementary file 6 — Additional file 6: Figure S3. Estimated genome size (Mb) versus GC content of all Black Sea MAGs retrieved in this work. Shape indicates the depth at which the MAG was recovered. MAGs are color-coded at the phylum level. [file 40793_2021_374_MOESM6_ESM.pdf]

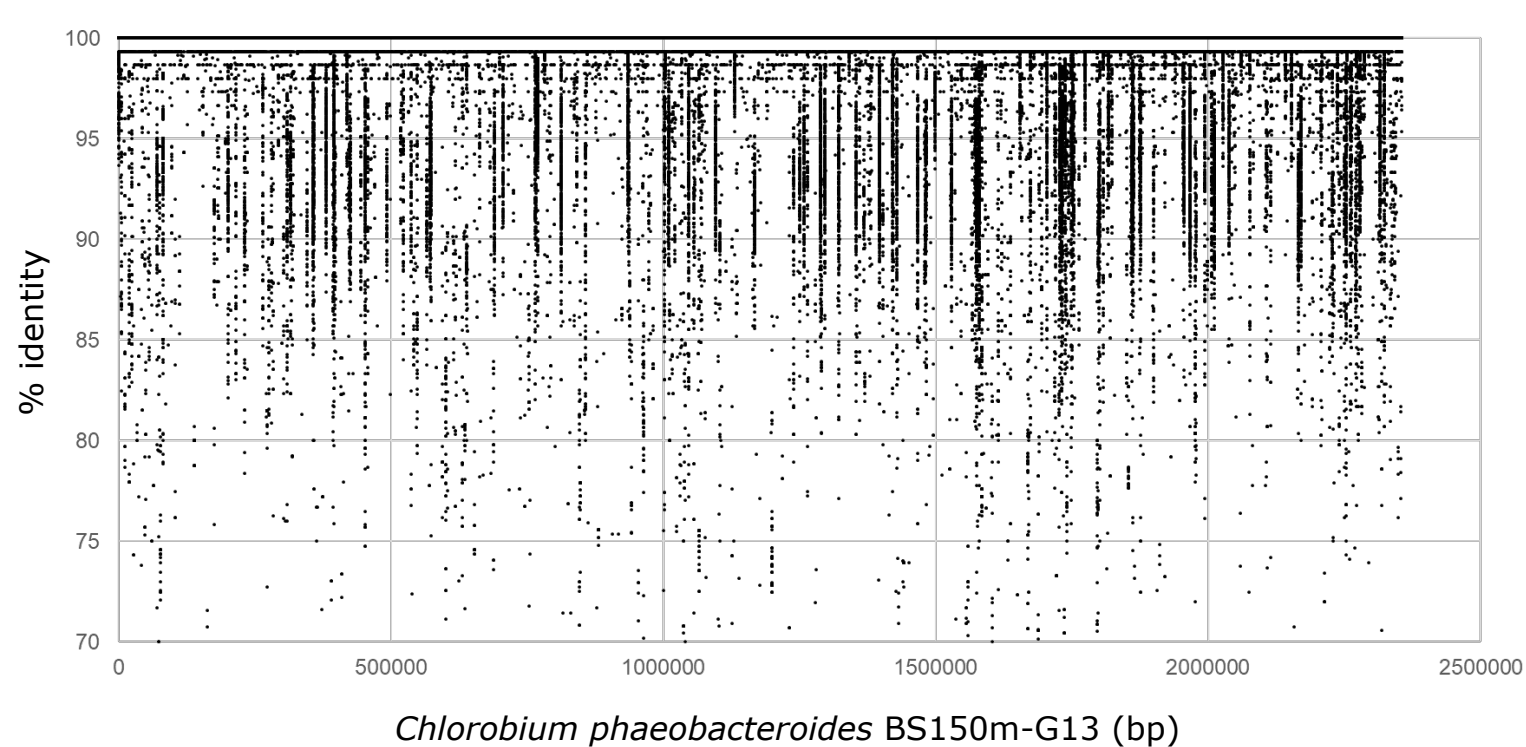

Supplement: Supplementary file 7 — Additional file 7: Figure S4. Recruitment plot of Chlorobium phaeobacteroides BS150m-G13 from the Black Sea 150 m redoxcline metagenome. Each dot represents a mapped read at > 95% of identity and > 50 bp of alignment lengths. [file 40793_2021_374_MOESM7_ESM.pdf]

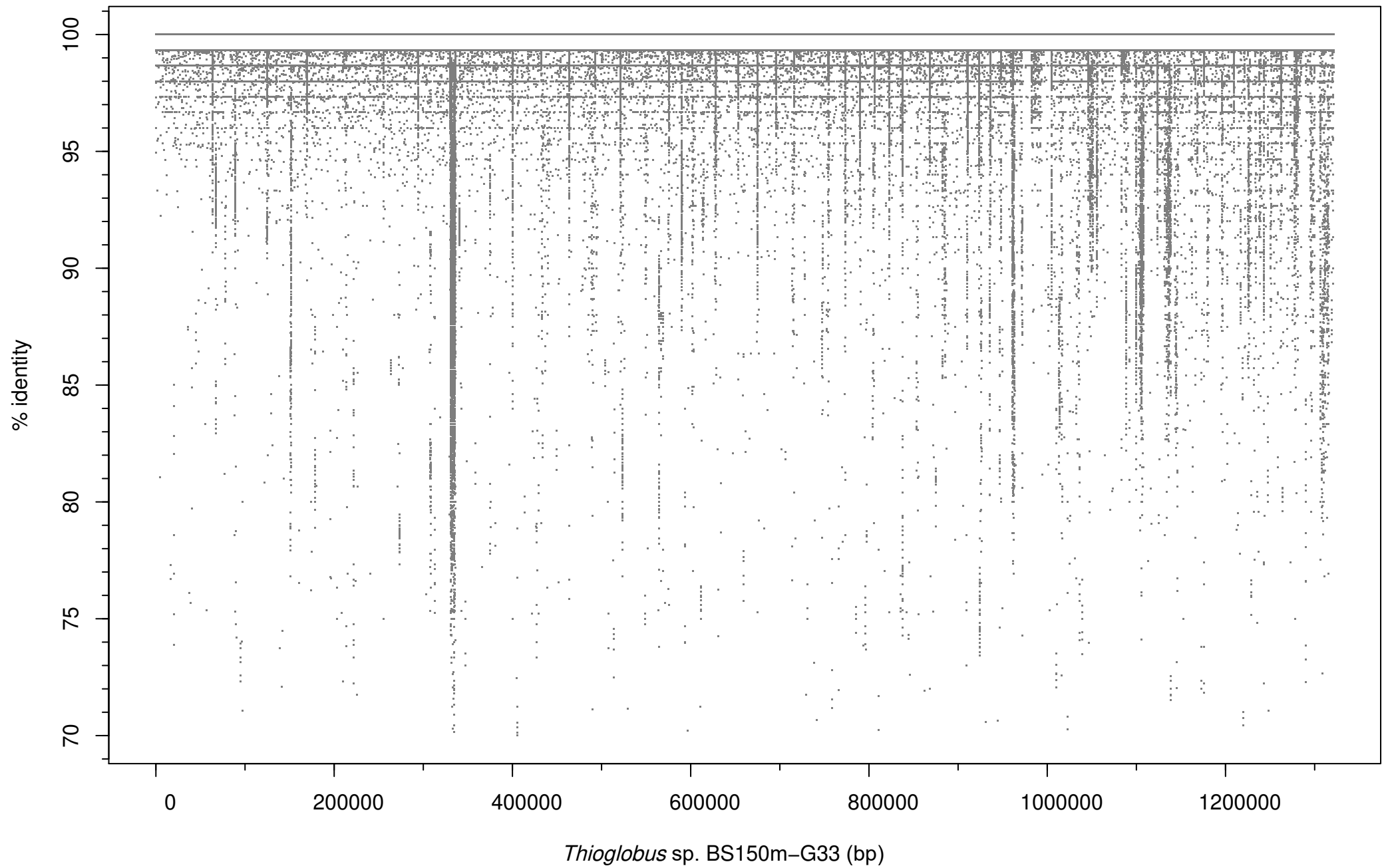

Supplement: Supplementary file 8 — Additional file 8: Figure S5. Recruitment plot of Thioglobus sp. BS150m-G33/G29 from the Black Sea 150 m redoxcline metagenome. Each dot represents a mapped read at > 95% of identity and > 50 bp of alignment lengths. [file 40793_2021_374_MOESM8_ESM.pdf]
